# GPX1 Localizes to the Nucleus in Prostate Epithelium and its Levels are not Associated with Prostate Cancer Recurrence

**DOI:** 10.3390/antiox7110167

**Published:** 2018-11-18

**Authors:** Dede N. Ekoue, Emmanuel Ansong, Lenny K. Hong, Larisa Nonn, Virgilia Macias, Ryan Deaton, Rawan Rupnow, Peter H. Gann, Andre Kajdacsy-Balla, Alan M. Diamond

**Affiliations:** 1 Department of Pathology, University of Illinois at Chicago, Chicago, IL 60612, USA; Dede.Ekoue@UTSouthwestern.edu (D.N.E.); eason2@uic.edu (E.A.); lennyh@uic.edu (L.K.H.); lnonn@uic.edu (L.N.); vmacias@uic.edu (V.M.); rdeaton@uic.edu (R.D.); pgann@uic.edu (P.H.G.); aballa@uic.edu (A.K.-B.); 2 Department of Epidemiology and Biostatistics, School of Public Health, University of Illinois at Chicago, Chicago, IL 60612, USA; ralloz2@uic.edu

**Keywords:** glutathione peroxidase, prostate, selenium, prostatectomy

## Abstract

Glutathione peroxidase 1 (GPX1) is an extensively studied selenium-dependent protein that reduces hydrogen and lipid peroxides to water. Because of its antioxidant function and its responsiveness to dietary intakes of selenium, an essential trace element whose levels are inversely associated with prostate cancer risk, GPX1 levels were assessed in a prostate cancer tissue microarray, comparing cases of recurrent prostate cancer following prostatectomy to non-recurrent controls. While GPX1 is generally considered as a protein that resides in both the cytoplasm and mitochondria, we detected strong nuclear staining by immunofluorescence using GPX1-specific antibodies. Nuclear localization of GPX1 was also observed in both primary prostate epithelial cells and the immortalized prostate-derived cell line RWPE-1, but not in LNCaP or PC3 prostate tumor-derived cell lines. Quantification of GPX1 levels in the entire cell, the cytoplasm, and the nucleus did not indicate any association of either its levels or subcellular distribution with prostate cancer recurrence. While GPX1 levels may not have an impact on survival among men with prostate cancer, the data indicates that this extensively characterized protein may have a novel function in the nucleus of prostate epithelial cells.

## 1. Introduction

Glutathione peroxidase 1 (GPX1) is a member of a class of proteins that are referred to collectively as selenoproteins, that contain selenium in the form of selenocysteine [1,2]. Selenocysteine is incorporated into these protein in response to an in-frame UGA codon in a process that requires a selenocysteine-specific transfer RNA (tRNA), dedicated translation factors, and a regulatory sequence (SECIS element) located in the 3’ UTR (untranslated region) of the corresponding mRNAs [3]. Among the members of this family are several related enzymes that are encoded by different genes with distinct expression patterns, each involved in the detoxification of lipid or hydrogen peroxides using reducing equivalents from glutathione [4]. Among these, GPX1 is the best characterized, having been initially described as an erythrocyte antioxidant enzyme [5]. Subsequent work showed that GPX1 levels are responsive to selenium availability, and that reduced GPX1 levels may be responsible for some of the pathologies associated with reduced dietary selenium intake [6]. While the knockout of *Gpx1* in mice does not have an overt phenotype unless challenged with oxidative stress [7], the results of human genetic studies have implicated GPX1 in a variety of diseases, due to the association of specific genetic variations, including a single nucleotide polymorphism at codon 198 and a variable number of repeated alanine-encoding codons and disease risk [6].

While not all studies have shown an inverse association between selenium status and prostate cancer incidence or risk, the results of meta-analyses have indicated that low selenium is likely a risk factor [8,9]. Prostate cancer is a prevalent disease, and many of the men who undergo prostatectomy did so unnecessarily, due to the difficulty in distinguishing indolent from aggressive cancers. Given the need for better ways of predicting prostate cancer outcomes, the anti-oxidant function of GPX1 and its responsiveness to selenium availability, we examined whether the levels of GPX1 in human prostate tissue were associated with recurrent cancer among men who were treated for their disease following prostatectomy using a tissue microarray that is specifically designed to test this relationship.

## 2. Materials and Methods 

### 2.1. Cell Culture and Imaging 

Human LNCaP and RWPE-1 prostate cell lines were obtained from ATCC (Manassas, VA, USA) and authenticated by analyzing 15 autosomal short tandem repeat loci and the sex-specific amelogenin locus to identify gender (Genetica DNA Laboratories, Burlington, NC, USA). Human primary prostate epithelial cells obtained from tissue after prostatectomy were prepared as previously described [10]. Confocal microscopy of indicated cells was performed as previously described [11].

### 2.2. Source of Clinical Samples

Prostate cancer outcome tissue microarrays (TMAs) were obtained from the Cooperative Prostate Cancer Tissue Resource (CPCTR), a multi-institutional consortium formed to bank prostatectomy tissue, with detailed uniform annotations of patient demographics, surgical pathology data, and follow-up history [12,13]. The TMAs used in this study included prostate cancer tissue cores of 0.6 mm diameter in quadruplicate, from 200 men (“cases”) who experienced biochemical recurrence and 200 non-recurrent controls matched by age at surgery (+/− 5 years), year of surgery, race, Gleason sum, and pathological stage. Biochemical recurrence is defined as a single post-surgery prostate specific antigen (PSA) value of above 0.4 ng/mL or two consecutive PSAs above 0.2 ng/mL). 

### 2.3. Immunohistochemistry 

Immunohistochemistry for GPX1 was performed by the University of Illinois at Chicago Research Histology and Tissue Imaging Core as previously described [11]. Briefly, deparaffinization and antigen retrieval (Ph 6; 20 min) was performed online using a Leica Bond-RX autostainer (Leica Biosystems, Wetzlar, Germany). The sections were washed with Bond Dewax solution (Leica Biosystems, AR9222, Buffalo Grove, IL, USA) at 72 °C, followed by a 100% ethanol wash. Slides were subsequently washed with bond wash solution, and target antigens were unmasked by incubation in Bond ER 1 Solution (pH 6) for 20 min at 100 °C (Leica Biosystems, AR9640, Buffalo Grove, IL, USA). Following a last wash, slides were incubated with a primary antibody for GPX1 (Rabbit polyclonal, Abcam # ab22604, 1:250 dilution), incubated with a secondary antibody, and processed as described [11]. The specificity of the antibody was verified by Western blotting, where a single 25 kDa band was observed in protein extracts derived from MCF-7 human breast cancer cells transfected with a GPX1 expression construct, but not in the parental GPX1 null cells, as well as blocking during immunohistochemistry (IHC) with a GPX1 antigenic peptide (Abcam # ab2530) as shown in Figure S1.

### 2.4. VECTRA Quantitative Imaging Analysis

The TMA slides were scanned at 20× magnification, spectrally unmixed, and the auto-fluorescence was removed. Each epithelial cell was digitally segmented into nuclear and cytoplasmic compartments using inForm® software (Perkin Elmer, Waltham, MA, USA) to define the border of each nucleus based on 4’,6’-diamidino-2-phenylindole (DAPI) staining and then the cytoplasmic pixels were sampled around each nucleus. After the manual exclusion of poor quality and benign glands/cores, the levels and locations of GPX1 were quantified in both subcellular compartments using the sum of fluorescent intensity across the relevant pixels.

### 2.5. Data Analysis 

Nuclear and cytoplasmic GPX1 intensities were assessed for normality, and log-transformed for statistical analysis. Patients were grouped into three categories based on their Gleason grade (Category 1 = Gleason ≤ 6, Category 2 = Gleason 7(3 + 4) Category 3 = Gleason 7(4 + 3) or ≥ 8). Patient tissues were also assigned to quartiles based on GPX1 intensity. Conditional logistic regression models were fitted to estimate the odds ratios and 95% confidence intervals for the risk of biochemical recurrence for each quartile of GPX1. The conditional models incorporated adjustments for case-control matching variables: Gleason grade, stage, and age at diagnosis; additional models were fit with pre-surgical PSA as a covariate, since PSA was not a matching factor. 

## 3. Results

### 3.1. GPX1 is a Nuclear Protein in Benign Human Prostate Epithelial Cells

**Figure 1:**
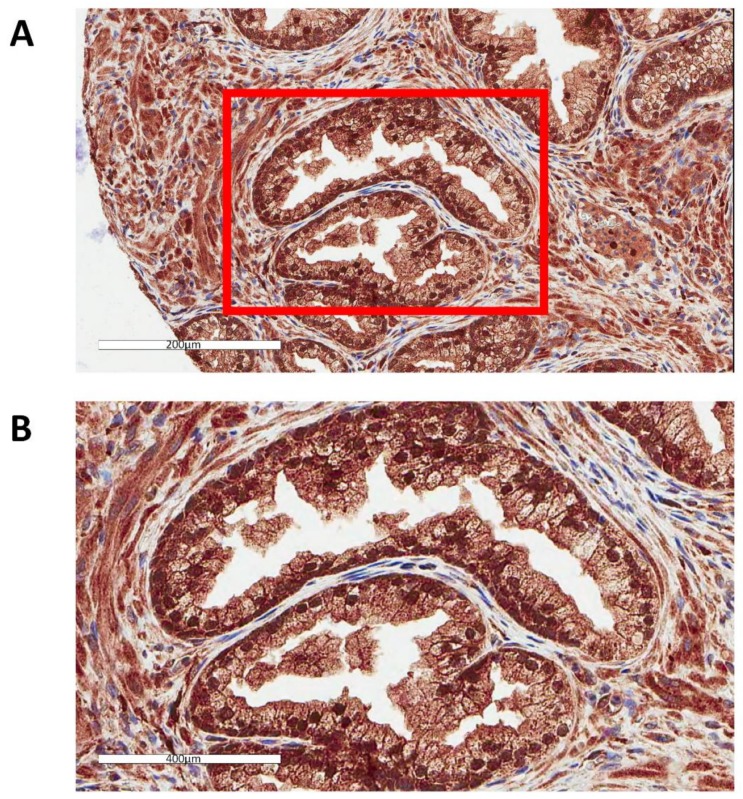
Localization of GPX1 in the nuclei of prostate epithelial cells. (**A**) Representative image of human prostatic tissue immunostained with anti-GPX1 antibodies, including highlighted portion of the upper image at a higher magnification (**B**).

**Figure 2:**
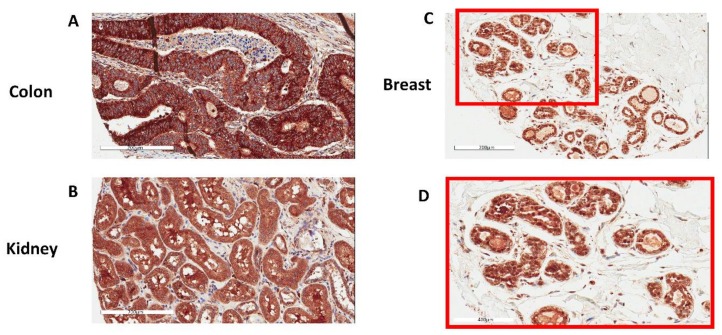
Representative images of GPX1 in human tissues immunostained with anti-GPX1 antibodies. (**A**) Colon, (**B**) Kidney, (**C**,**D**) Breast with panel (**D**) being a higher magnification of the inset in (**C**).

GPX1 has been extensively studied in many tissues and cell types, but the protein has not been visualized in human prostate tissue. To accomplish this, GPX1 was examined in prostate tissue by IHC (Figure 1). The tissue was part of a TMA in which many cores contained both tumors and benign glands, as determined by a pathologist. Surprisingly, the major staining of benign glands with anti-GPX1 antibodies was in the nucleus of most, if not all of the prostate epithelial cells. Using the same antibodies, other tissues were examined to identify the cellular location of GPX1. As seen in Figure 2, nuclear staining was also observed in breast tissue. In contrast, nuclear staining was not observed in either the colon or kidney (Figure 2). 

### 3.2. Nuclear Localization of GPX1 in Immortalized Human Prostate Cells but Not in Tumor-Derived Cell Lines

**Figure 3:**
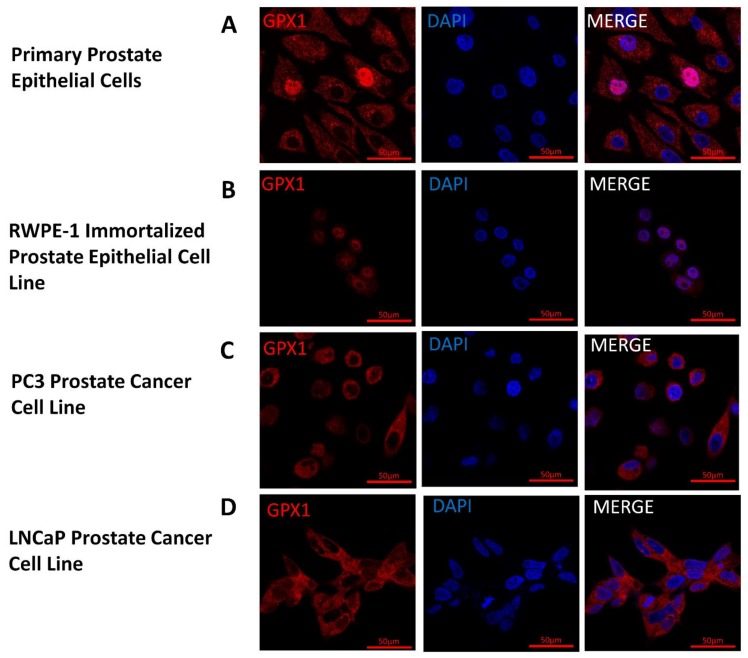
Location and levels of GPX1 in cultured cells. GPX1 appears in red and nuclei are indicated by staining with 4’,6-diamidino-2-phenylindole (DAPI) in blue. (**A**) Primary Prostate Epithelial Cell; (**B**) RWPE-1 Immortalized Prostate Epithelial Cell Line; (**C**) PC3 Prostate Cancer Cell Line; (**D**) LNCaP Prostate Cancer Cell Line.

Given the unexpected location of GPX1 in the nucleus of prostate epithelium, the subcellular location of GPX1 was determined by immunofluorescence in primary prostate cells, the RWPE-1 immortalized prostate epithelial cell line, and the LNCaP and PC3 human prostate cancer-derived cell lines (Figure 3). GPX1 localized to the nucleus of immortalized RWPE-1 cells and primary prostate cells, although the staining was sporadic with under 5% of the observed cells exhibiting nuclear GPX1 staining. In contrast, GPX1 was localized to the cytoplasm of LNCaP and PC3 human prostate cancer-derived cells with no apparent nuclear staining (Figure 3).

### 3.3. Neither Levels nor Cellular Location of GPX1 in Human Prostate Epithelium are Associated with Prostate Cancer Recurrence

Given the data presented above indicating nuclear localization of GPX1 in primary or immortalized prostate cells but not in cancer-derived cell lines, we assessed whether GPX1 levels or sub-cellular location was associated with prostate cancer recurrence following a prostatectomy. This was done by quantifying the GPX1 levels in tissue cores obtained from 200 prostate cancer patients who experienced biochemical recurrence (rising PSA levels) and 200 matched control patients whose cancers did not return, by immunohistochemistry. Levels of GPX1 were segregated into quartiles, and the odds ratios of experiencing a recurrence of prostate cancer were presented in Table 1. There was no association between GPX1 levels either in the entire cell, in the cytoplasm, or in the nucleus, with stage, grade, or biochemical recurrences.

## 4. Discussion

Several lines of evidence have indicated that GPX1 may be relevant to prostate cancer etiology. GPX1 levels are responsive to selenium availability and previous studies have reported an inverse relationship between selenium nutritional status and prostate cancer risk [8]. The known enzymatic function of GPX1 in the detoxification of lipid and hydrogen peroxides, was also considered as potentially being beneficial in reducing reactive oxygen species that could damage DNA and other biomolecules. For these reasons, GPX1 was imaged in prostate tissue and cell lines, and it was assessed whether GPX1 levels were associated with prostate cancer recurrence after prostatectomy.

This is the first study to our knowledge that examined whether prostate cancer recurrence was associated with levels of the GPX1 protein. A search of the Oncomine database [14] revealed 14 studies that compared the levels of GPX1 transcripts between normal prostate and tumor tissues, and none revealed a significant association. Several studies have examined whether polymorphisms in the *GPX1* gene were associated with prostate cancer risk, and the results have been mixed. While most studies have not detected a significant association between *GPX1* polymorphisms and the risk of prostate cancer or cancer recurrence [15,16,17,18,19,20,21,22], two studies with large sample sizes reported an association between *GPX1* polymorphisms and the risk of advanced disease [23,24], while another study reported on the association between a trinucleotide repeat variation in the coding region of GPX1 and young onset prostate cancer [25]. GPX1 is highly regulated at the level of translation by selenium availability and the readthrough of the in-frame UGA triplet that is the codon for selenocysteine. Therefore, we investigated whether the levels of GPX1 were associated with prostate cancer recurrence in a TMA that was specifically designed to assess this endpoint, with the results indicating no difference in GPX1 levels between recurrent and non-recurrent cases.

An unexpected result was the strong staining of GPX1 in the nuclei of both benign prostate tissues and immortalized cells, but not in cancer cells. While there have been extensive reports of GPX1 as a cytoplasmic or mitochondrial protein, some early reports have indicated the nuclear location of GPX1 in rat hepatocytes—either by the detection of enzyme activity or immunodetection, which may have been due to detection of the closely related GPX family member, GPX2 [26,27,28], with one report indicating the identification of GPX in the nucleus of normal rat hepatocytes was likely to be an artifact [29]. While one published report indicated diffuse and nuclear GPX detection in basal cells of the prostate with trace detection in epithelial cells [30], there were uncertainties as to the specificity of the antibodies used in that study [31]. How GPX1 enters the nucleus of prostate epithelial cells is unknown. A search for nuclear localization sequences (NLS) among the 25 human selenoproteins using cNLS Mapper, a program that is specifically designed to recognize mono- and bi-partite NLS, revealed possible NLS in 15 selenoproteins, including GPX2 and GPX4, but not GPX1 [32]. As a tetramer of molecular weight of approximately 100 kDa, GPX1 is too large to freely diffuse into the nucleus [33], raising the possibility that GPX1 enters the nucleus as a monomer, or by a yet-to-be-resolved transport mechanism. The stoichiometry of nuclear GPX1, and consequently its function in human prostate tissue, remains an area of future investigation.

## Figures and Tables

**Table 1:** For each individual, the average of the total GPX1 level per cell was calculated.

Conditional Logistic Regression of Prostate Cancer Recurrence GPX Analysis		
	Odds Ratio (95% CI)	*p*-Value ^1^
**GPX in Whole Cell**		
Second Quartile	0.980 (0.501,1.916)	0.9519
Third Quartile	0.671 (0.338,1.332)	0.2541
Fourth Quartile	0.876 (0.446,1.724)	0.7025
**GPX in Cytoplasm**		
Second Quartile	0.976 (0.506,1.883)	0.9423
Third Quartile	0.946 (0.471,1.903)	0.8773
Fourth Quartile	1.076 (0.560,2.067)	0.8252
**GPX in Nucleus**		
Second Quartile	0.617 (0.320,1.188)	0.1487
Third Quartile	0.499 (0.240,1.037)	0.0625
Fourth Quartile	0.747 (0.386,1.445)	0.3862

Individuals were matched by race, Gleason sum score, PT, PN, PM, age and year of surgery. ^1^ Odds ratios and p-values are from conditional logistic regression of prostate cancer recurrence and were adjusted for prostate specific antigen (PSA) levels. The reference group for all predictors is the first quartile.

## Supplementary Materials

The following are available online at http://www.mdpi.com/2076-3921/7/11/167/s1, Figure S1: Specificity of the GPX1 antibody was confirmed with a blocking peptide.
