# Supplementary figures and images for: GPX1 Localizes to the Nucleus in Prostate Epithelium and its Levels are not Associated with Prostate Cancer Recurrence
Source: Antioxidants (Basel). 2018 Nov 18;7(11):167. doi: 10.3390/antiox7110167 (PMC6262378; doi:10.3390/antiox7110167)

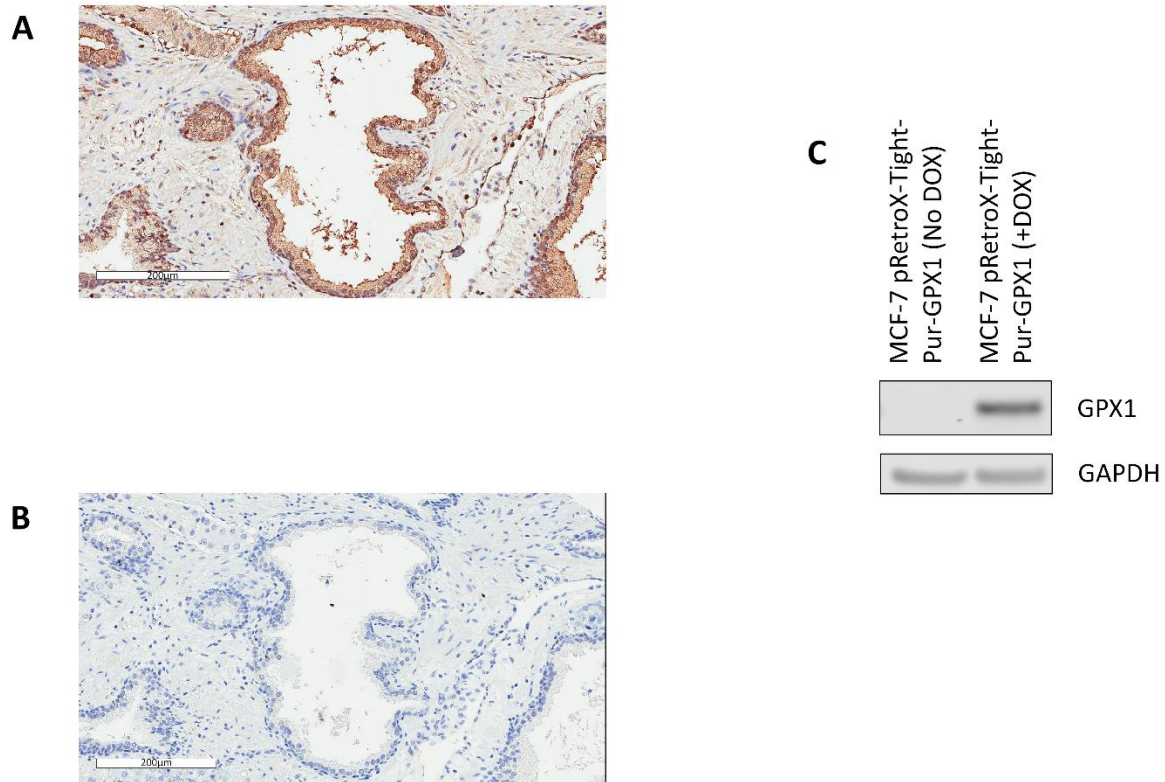

Figure S1: Specificity of the GPX1 antibody was confirmed with a blocking peptide

Supplement: Supplementary file 1 [file antioxidants-07-00167-s001.pdf]
